# Thermodynamic basis of the *α*-helix and DNA duplex

**DOI:** 10.1007/s00249-021-01520-w

**Published:** 2021-04-24

**Authors:** A. I. Dragan, C. Crane-Robinson, P. L. Privalov

**Affiliations:** 1 Institute of High Technologies, Taras Shevchenko National University of Kyiv, Kyiv, 01601 Ukraine; 2 Biophysics Laboratories, School of Biology, University of Portsmouth, Portsmouth, PO1 2DT UK; 3 Department of Biology, Johns Hopkins University, Baltimore, MD 21218 USA

**Keywords:** *α*-Helix, DNA double helix, Stability, Van der Waals interactions, Hydrogen bonding

## Abstract

Analysis of calorimetric and crystallographic information shows that the α-helix is maintained not only by the hydrogen bonds between its polar peptide groups, as originally supposed, but also by van der Waals interactions between tightly packed apolar groups in the interior of the helix. These apolar contacts are responsible for about 60% of the forces stabilizing the folded conformation of the α-helix and their exposure to water on unfolding results in the observed heat capacity increment, i.e. the temperature dependence of the melting enthalpy. The folding process is also favoured by an entropy increase resulting from the release of water from the peptide groups. A similar situation holds for the DNA double helix: calorimetry shows that the hydrogen bonding between conjugate base pairs provides a purely entropic contribution of about 40% to the Gibbs energy while the enthalpic van der Waals interactions between the tightly packed apolar parts of the base pairs provide the remaining 60%. Despite very different structures, the thermodynamic basis of α-helix and B-form duplex stability are strikingly similar. The general conclusion follows that the stability of protein folds is primarily dependent on internal atomic close contacts rather than the hydrogen bonds they contain.

## Introduction

**Fig. 1:**
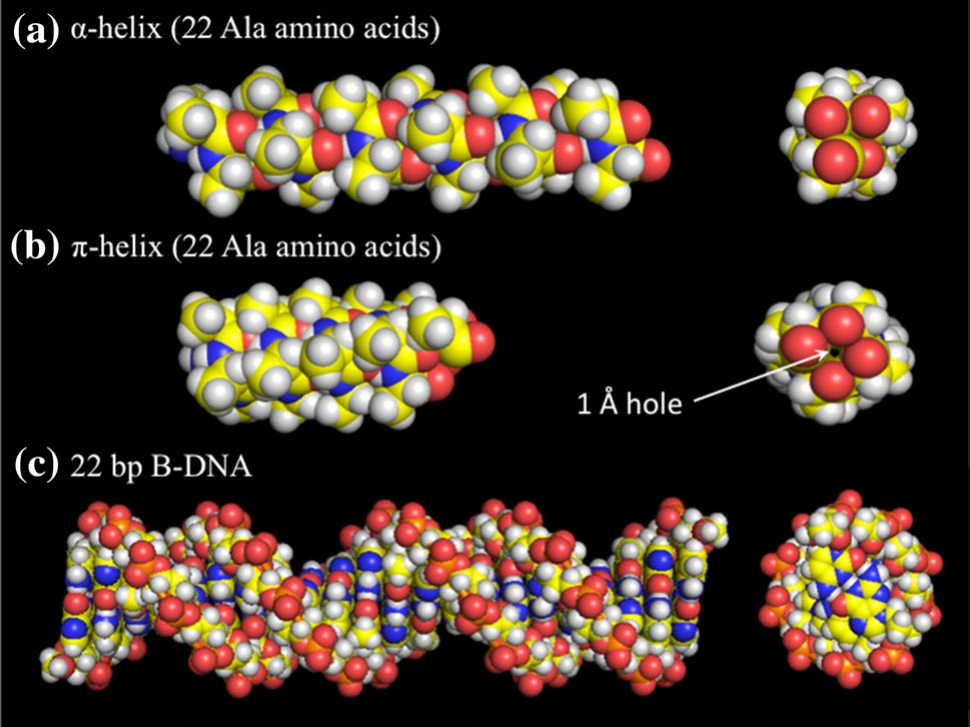
Space filling models of the α-helix, П-helix and B-form DNA duplex. Only for the П-helix is there any central space remaining in the structure

The first regular conformation of the polypeptide chain to be recognised, the α-helix, was proposed by Pauling et al. (1951) and it appeared to be held together largely by hydrogen bonds resulting from association of the positively charged amino hydrogen of the *N*th residue with the negatively charged carbonyl oxygen of the (*N* + 4)th residue along the polypeptide chain. In the less stable П-helix, the hydrogen bond connects with the (*N* + 5)th residue, with a resulting central axial space (see Fig. 1). In the disordered state of the polypeptide chain, the groups participating in this intramolecular H-bond are expected to be hydrogen bonded to water molecules, so it was difficult to envisage the source of any enthalpic stabilization of the helix from H-bonding. In contrast, formation of the helix must be accompanied by the shedding of water molecules from the amino and carbonyl groups into the bulk solvent, resulting in a substantial entropy increase. So what is the evidence that—in aqueous solution—intramolecular hydrogen bonds are entropic rather than enthalpic interactions?

With the definition of the DNA double helix by Watson and Crick (1953), it appeared that its regular structure is also maintained by hydrogen bonding, in this case between the conjugate base pairs: A with T and G with C (see Fig. 1). To investigate the physical basis of these two regular conformations, the α-helix of polypeptide chains and the DNA duplex, various physical methods have been used: optical, hydrodynamic, crystallographic and scanning microcalorimetry (DSC), the last specially developed for this purpose (see Privalov 2012 for an overview). Importantly, DSC shows that heating of both the α-helix and the DNA double helix results in melting their regular conformation and this proceeds with substantial heat absorption accompanied by a significant heat capacity increment. To make comparison between the two structures, the thermodynamic signatures of both the α-helix and the DNA duplex need to be defined.

## Calorimetry of the *α-*helix

**Fig. 2:**
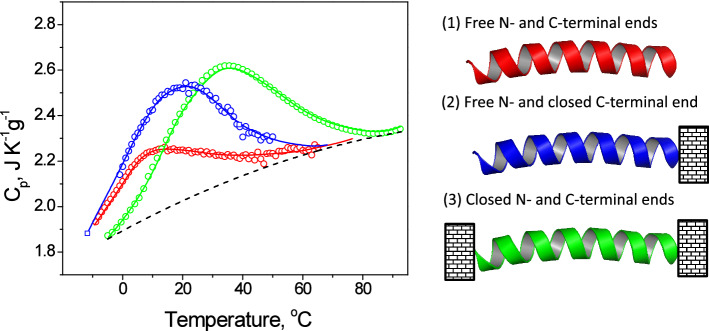
The partial specific heat capacity functions of the isolated basic segment of GCN4 with (1) free ends, red line; (2) attached at the C-terminal end to the leucine zipper, blue line (Dragan et al. 2004a); (3) the basic segment with two covalently closed terminal loops, green line (Taylor et al. 1999)

Early studies of the *α-*helix were carried out using isolated single helices from sperm whale myoglobin. However, the temperature induced unfolding of such helices is not a two-state transition because upon heating they gradually melt from both ends. Therefore, in our experiments, we used a single helix from the basic segment of the yeast bZIP protein GCN4, having covalently closed terminal loops attached at each end to block end-fraying (Taylor et al. 1999; Dragan et al. 2004a). This polypeptide is 100% helical and unfolds/refolds in an all-or-nothing fully reversible manner, absorbing/releasing considerable heat (Fig. 2). Modelling the *C*_p_/*T* function for melting this 29 amino acid polypeptide (the green curve in Fig. 2) as a 2-state process shows the best fit is for an enthalpy, ∆*H*, of 69 kJ/mol-peptide and a heat capacity increase, ∆*C*_p_, of 0.46 kJ/K.mol-peptide, i.e. ∆*H*=2.4 kJ/mol-amino acid and ∆*C*_p_ = 0.016 kJ/K.mol-amino acid. Other studies of individual *α-*helices (e.g. Richardson et al. 1999; Lopez et al. 2002) led to somewhat higher enthalpy values of ~ 3.5 kJ/mol-amino acid but no increase in heat capacity was observed. Calorimetric data obtained for *α-*helices raise the question as to the source of the enthalpy and its temperature dependence, as well as the basis of its thermodynamic stability.

## Analysis of structural data for α-helices

Unfolding of an *α-*helix results in exposure of apolar and polar groups and this can give rise to a heat capacity change in consequence of altered hydration, i.e. an enthalpy dependence on temperature. The increase in accessible surface area, ∆ASA, of both polar and apolar surface, that becomes exposed on unfolding several *α-*helices was determined using the NACCESS programme and these increases then used to predict the consequent heat capacity change, ∆*C*_p_, calculated using the equation proposed by Makhatadze and Privalov (1995):

1 $$ \Delta C_{{\text{p}}}  = 2.14 \times \Delta {\text{ASA}}^{{{\text{apolar}}}}  - 1.27 \times \Delta {\text{ASA}}^{{{\text{polar}}}}, $$

where ∆ASA values are in Å^2^ and the coefficients typically in J/K/Å^2^.

This equation reflects the fact that exposure of apolar surface—meaning its hydration—leads to an *increase* in the heat capacity (positive ∆*C*_p_), whereas exposure of polar surface results in a decreased heat capacity (negative ∆*C*_p_).

The first entry in Table 1 gives data for a fully folded ‘all-*α*’ protein: sperm whale myoglobin (swMb). It consists of 8 tightly packed helices and the interior is very rich in non-polar contacts—relative to polar contacts—with the result that the heat capacity *increases* very substantially on melting. The predicted ∆*C*_p_ is in good agreement with the experimentally measured value of 93 J/K.mol-residue, indicating the reliability of Eq. (1).

**Table 1:** Analysis of changes in solvent accessible areas, ASAs, (polar and apolar), upon unfolding an intact protein and three alpha-helices

Object	Polarity	Calculated data					Experimental data	
	ASA polarity	ASA (folded) Å^2^	ASA (unfolded) Å^2^	ΔASA Å^2^	Overall Δ*C*_p_, kJ/(K.mol)	Δ*C*_p_, J/(K.mol-residue)	Overall Δ*C*_p_, kJ/(K.mol)	Δ*C*_p_, J/(K.mol-residue)
swMb: intact sperm whale myoglobin, 153 aa	Apolar	4928	13,110	8182	14.4	93.5	14	93
	Polar	2980	5431	2451				
*α*-Helix generated from complete swMb sequence	Apolar	10,853	13,110	2257	1.92	12.47	NK	NK
	Polar	3140	5431	2291				
*α*-Helix from the 29 a.a. GCN4 sequence from Taylor et al. (1999)	Apolar	1246	1622	376	0.24	12.7	0.46	16
	Polar	908	1350	442				
*α*-Helix from the Lpp56 sequence Dragan et al. (2004b)	Apolar	3567.4	4707.4	1140	0.94	16.78	0.83	14.8
	Polar	1629.2	2810.3	1181				

Calculation of corresponding unfolding heat capacities and comparison with experimental data

Heat capacity changes calculated using Eq. 1 (see Makhatadze and Privalov 1995) based on changes in Accessible Surface Area (∆ASA), obtained using NACCESS, for a single intact protein (sperm whale myoglobin) and three *α*-helices generated in silico

*NK* not known

The remaining three entries in Table 1 are individual helices with *α-*helical coordinates generated in silico. The first is the complete sequence of swMb configured as one continuous *α-*helix, the second is the fully helical 29 residue monomeric polypeptide studied calorimetrically by Taylor et al. (1999) and the third is the 56 residue sequence of Lpp56, the unit of the trimeric *α-*helical coiled-coil from the outer membrane of *E. coli*, studied by Dragan et al. (2004b). For all three *α-*helices unfolding leads to exposure of similar total areas per amino acid, amounting to ~ 30 Å^2^. Furthermore, the areas of apolar and polar surface exposed are approximately equal and since the apolar coefficient in Eq. (1) is significantly greater than the polar coefficient, the prediction is a net positive value of ∆Cp, calculated as + (12–17) J/K.mol-residue for these three helices. Strikingly, these values are close to those measured experimentally for the GCN4-derived polypeptide of Taylor et al. (1999), ∆*C*_p_ = 16 J/K.mol-residue, and the Lpp56 monomer from Dragan et al. (2004b), ∆*C*_p_ = 14.8 J/K.mol-residue. The modelling of these *α-*helices in silico therefore gives a good representation of their structure in solution and provides an understanding of why the heat capacity change on melting the *α-*helix has a small positive value.

The dominance of the first term of Eq. (1) shows that the van der Waals interactions between tightly compacted apolar atoms are the prime contributor to *α-*helix stabilization. If the H-bonds were the principle agent of helix stability then the exposure of their polar surface would be expected to dominate ∆ASA and the heat capacity change on unfolding consequently be negative. But this is not observed: the heat capacity change is positive, i.e. the stabilizing enthalpy comes principally from the hydrophobic interactions.

## Comparing the thermodynamic signatures of the *α-*helix and the DNA duplex

**Fig. 3:**
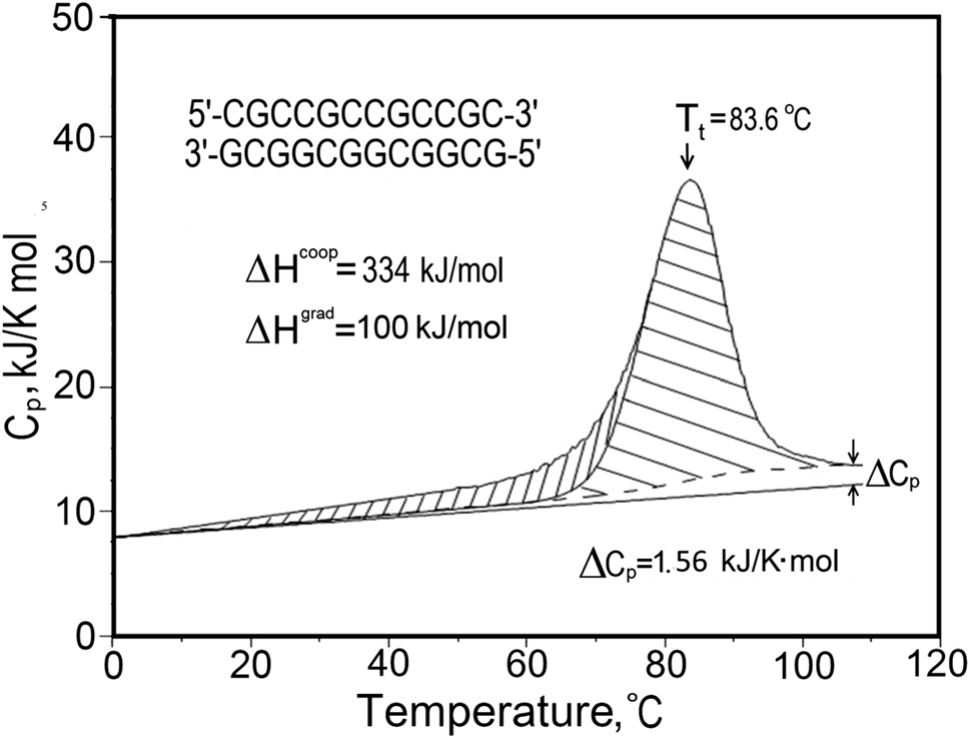
The observed heat capacity profile of a 12 bp all-CG duplex. The experimental excess heat effect is deconvoluted into the non-cooperative (gradual, vertical hatching) and cooperative (horizontal hatching) phases (see Vaitiekunas et al. 2015)

The substantial heat effect (∆H) and its significant dependence on temperature (∆Cp) for melting the DNA duplex have recently been investigated in detail using short synthetic duplexes (Vaitiekunas et al. 2015; Dragan et al. 2019; Privalov and Crane-Robinson 2020), see Fig. 3. This allowed determination of the thermodynamic parameters characteristic of the dissociation of CG and AT pairs. After making allowance for the loss of minor groove hydration to the AT pairs, it was found that the intrinsic melting enthalpy of both pairs is essentially the same. Since the CG pair carries an extra H-bond relative to AT, it follows that H-bonding is not contributing to the observed enthalpies. As regards the overall entropy of dissociation, which has a large positive conformational contribution, the more positive net value observed for the AT pair (by 4 J/K.mol-bp) reflects a lower negative contribution to ∆*S* from AT hydration as compared to CG hydration: – 4 J/K.mol-bp is thus the entropy decrease from hydration on dissociating a single H-bond.

The observation that dissociation of H-bonds in DNA melting is non-enthalpic accords with the expectation that the process is an exchange of H-bonding, not just the loss of base pairing. Moreover, the purely entropic nature of H-bond disruption—in water solution—is not unexpected as H-bonds are essentially electrostatic contacts, interactions experimentally demonstrated to be entirely entropic. For example, in protein/DNA complexes the ionic binding of negative phosphates and positive sidechains of lysine and arginine means that the affinity is strongly reduced by increased ionic strength. However, it is observed that the enthalpy of the protein/DNA interaction is independent of the salt concentration (see Dragan et al. 2004c; Privalov et al. 2011), showing that the ionic links are indeed non-enthalpic. The drop in affinity with increasing ionic strength results entirely from the entropy of released counter-ions (principally cations from the DNA) being less at higher salt concentrations.

The conclusion therefore is that the contribution of H-bond pairing to DNA duplex stability is entirely entropic in nature, i.e. non-enthalpic. So what is the source of the calorimetrically observed enthalpy? The answer can only be that П–П stacking of base pairs is the main contributor, as expected from the observation of substantial enthalpy on melting single stranded oligo-A, a situation in which no internal H-bonding is expected (Breslauer and Sturtevant 1977).

Although both the *α-*helix and the duplex are rod-like macromolecules, the question arises as to whether they are supported by a similar combination of thermodynamic forces. In both cases, there is a substantial negative enthalpy driving formation of the folded structures and this is enhanced by the positive entropy derived from dehydrating the polar groups that form the H-bonds. In structural terms, base stacking in the duplex and close packing of atoms in the *α-*helix provide the driving enthalpy, while a purely entropic force is provided by the release of water molecules from the DNA bases and from the peptide atoms that form H-bonds in the *α-*helix. These two driving forces are, of course, opposed by the substantial reduction in conformational entropy on forming both structures.

Table 2 compares the key thermodynamic parameters for the base pairs of DNA with those determined for the 29-residue α-helix derived from GCN4. The data for the AT and CG pairs of DNA are taken from Privalov and Crane-Robinson 2020 and for the *α-*helix the 29-residue polypeptide of Taylor et al. 1999, all at the standard temperature of 25 °C—which is close to the melting temperature of this helix (*T*_m_ = 28 °C). Importantly, it is assumed that the thermodynamic parameters for forming the H-bond in the *α-*helix are the same as for the intermolecular H-bonds in the DNA duplex, i.e. non-enthalpic with an entropy increase of 4 J/K.mol H-bond, which provides an entropy factor (∆*T*∆*S*) of – 1.2 kJ/mol H-bond to the overall Gibbs free energy.

**Table 2:** Data for formation of the DNA duplex (Privalov and Crane-Robinson 2020) and for folding a 29 amino acid α-helix derived from GCN4 (Taylor et al. 1999; Dragan et al. 2004a)

All at 25 °C	DNA	DNA	α-HELIX
∆*H* kJ/mol-bp	− 19	− 19	– 2.31 kJ/mol.a.a.
∆*S* J/K.mol-bp	− 36.2	− 40.5	– 7.82 J/K.mol.a.a.
−*T*∆*S* kJ/mol-bp	+ 10.7	+ 11.9	+ 2.33 kJ/mol.a.a.
∆*G* kJ/mol-bp	− 8.3	− 7.1	+ 0.02 kJ/mol.a.a.
*T*∆*S* kJ/mol H-bond (assumed constant)	+ 1.2	+ 1.2	+ 1.2 kJ/mol H-bond
*−T*∆*S*^H−bonding^ kJ/mol-bp (total)	− 3.6	− 2.4	– 1.2 kJ/mol.a.a.
*−T*∆*S*^Conformational^ kJ/mol-bp	+ 14.3	+ 14.4	+ 3.53 kJ/mol.a.a.
*T*∆*S*^H−bonding^ /∆*G*	0.43	0.34	Not meaningful
*T*∆*S*^H−bonding^ /∆*H*	0.19	0.13	0.52

**Fig. 4:**
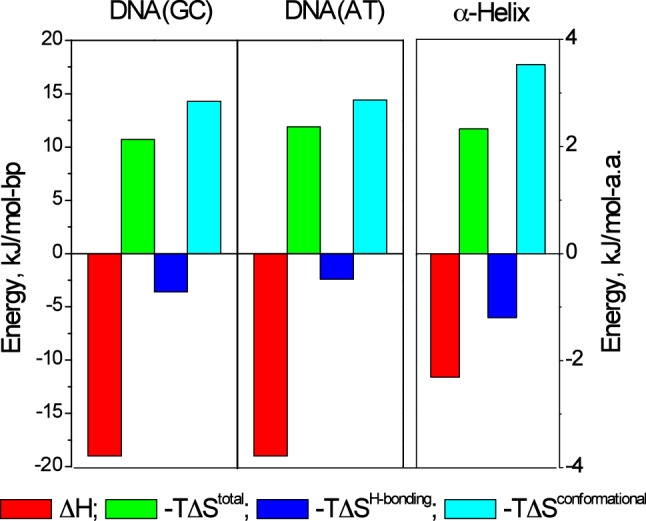
Enthalpies and entropies of forming the base pairs of the DNA duplex and folding the α-helix at 25 °C. Enthalpies in red. The total entropy factor (in green) is made up of a large reduction in conformational entropy (*T*∆*S*^conformational^ in cyan) and an increase in entropy from water release on forming the H-bonds (in dark blue)

The data in Table 2 show that the enthalpy/entropy pattern is similar for formation of the two structures. As illustrated in Fig. 4, the enthalpy is the dominant driving force (in red), counteracted by the large reduction in conformational entropy (in cyan) but the entropy increase from water release on forming the H-bonds (dark blue) makes a favourable contribution to folding both the duplex and the helix.

The last two rows of Table 2 quantify the relative contribution of entropic H-bonding to the overall stability of the two macromolecules. The first (*T*∆*S*^H-bonding^/∆*G*) is the H-bonding entropy factor as a proportion of the total Gibbs energy. This ratio is not meaningful for the α-helix used here as its Gibbs energy is so close to zero at 25 °C, but for the DNA base pairs, an averaged ratio of 0.4 implies that 40% of the Gibbs energy is entropic, i.e. 60% enthalpic.

The second ratio (*T*∆*S*^H-bonding^/∆*H*) is the H-bonding entropy factor as a proportion of the enthalpy, the other component of the driving force for folding, seen in Fig. 4 as the blue relative to the red bars. This ratio shows that for the duplex the entropic free energy derived from water release on forming the base pairs represents about 15% of the enthalpic free energy derived from base stacking and other van der Waals close contacts. For the α-helix, the H-bonding entropy factor gives about half as much free energy as does the enthalpic component derived from atomic close contacts, i.e. the H-bonds provide approximately 1/3rd of the total force driving helix folding.

## Conclusions

The data in Table 2/Fig. 4 demonstrate that the free energy driving folding of the α-helix and the DNA duplex comes from two sources, the entropy of releasing water bound to the polar groups that form H-bonds, which is the minor component, and the majority component, which is enthalpic. Thus although the two macromolecules are very different in their molecular composition and structure, they are similar as regards the forces driving their formation. For both polymers, although the hydrogen bonds are responsible for defining the specificity of the structures, it is the van de Waals forces between closely packed atoms that play the primary role in maintaining their overall stabilities.

The finding that for the α-helix—as with the DNA duplex—the entropy derived from forming internal H-bonds is of lower magnitude than the enthalpy, raises the question as to the overall role of H-bonding in protein folding and structure. Hydrogen bonds are involved in secondary structure elements in addition to the α-helix and in tertiary contacts, the ‘dehydration entropy’ of which should not differ greatly from that in the α-helix. The overall picture is clear from the surface areas dehydrated on folding. Whereas for the isolated α-helix the polar and apolar ∆ASAs are approximately equal, for completely folded domains such as swMb (see Table 1) ∆ASA^apolar^ is very much greater than ∆ASA^polar^, implying that the enthalpy of van der Waals contacts greatly exceeds the entropy factor derived from water release on forming H-bonds. This means that the concept of protein fold largely maintained by a network of H-bonds is not an appropriate model. The leading role in stabilizing protein folds is played by the van der Waals forces derived from internal close contacts.
